# Universal admission screening for COVID-19 using quantitative antigen testing and questionnaire screening to prevent nosocomial spread

**DOI:** 10.1371/journal.pone.0277426

**Published:** 2022-11-10

**Authors:** Masayo Morishima, Muneyoshi Kimura, Takashi Sakoh, Ryosuke Yamamuro, Sho Ogura, Namiko Takahashi, Masaru Baba, Takashi Herai, Shigeyuki Endo, Shuichi Taniguchi, Hideki Araoka

**Affiliations:** 1 Department of Infectious Diseases, Toranomon Hospital, Tokyo, Japan; 2 Department of Infection Control and Prevention, Toranomon Hospital, Tokyo, Japan; 3 Department of Clinical Laboratory, Toranomon Hospital, Tokyo, Japan; 4 Okinaka Memorial Institute for Medical Research, Tokyo, Japan; Menzies School of Health Research, AUSTRALIA

## Abstract

**Background:**

In this study, we investigated diagnostic accuracy of quantitative severe acute respiratory syndrome coronavirus 2 (SARS-CoV-2) antigen testing and whether universal screening was effective to prevent a nosocomial outbreak of coronavirus disease 2019 (COVID-19).

**Methods:**

All adult patients admitted to an acute-care hospital in Tokyo, Japan, after receiving LUMIPULSE SARS-CoV-2 Ag using a nasopharyngeal swab and a brief questionnaire to evaluate symptoms and exposures from December 3, 2020 to March 20, 2021 were included.

**Results:**

Of the 5191 patients, 53 were antigen-positive, 19 were inconclusive and 5119 were negative. The sensitivity and specificity (positive or inconclusive results) of the quantitative antigen test for COVID-19 diagnosis at admission was 0.957 (95% confidence interval [CI]: 0.855–0.995) and 0.995 (95% CI: 0.992–0.997), respectively. Six asymptomatic patients were identified on admission. Two patients were antigen-negative and diagnosed with COVID-19 later; however, they had been isolated prior to diagnosis because both had symptoms of COVID-19 and exposure. No nosocomial infections occurred during the period.

**Conclusion:**

Quantitative SARS-CoV-2 antigen testing was found to be valid for the early detection of asymptomatic COVID-19 patients as a universal screening test on admission.

## Introduction

It is challenging to prevent coronavirus disease 2019 (COVID-19) infection, because of severe acute respiratory syndrome coronavirus 2 (SARS-CoV-2) transmission from pre-symptomatic or asymptomatic patients [1]. Reports state that the peak viral load occurs between pre-onset and onset of illness [2]. To prevent nosocomial infection, early detection and isolation of infected patients are crucial [3]. Upon admission, some hospitals use screening tests, such as the SARS-CoV-2 nucleic acid amplification test (NAAT) [4–6], in addition to assessing symptoms and exposures.

The U.S. Center for Disease Control and Prevention (CDC) released interim guidance, including an algorithm for screening asymptomatic and symptomatic patients in community-based settings using antigen testing [7]. However, to the best of our knowledge, whether quantitative antigen testing is useful as the universal admission screening test in healthcare facilities has not yet been studied.

LUMIPULSE SARS-CoV-2 Ag is a two-step sandwich chemiluminescent enzyme immunoassay-based quantitative SARS-CoV-2 antigen test developed in Japan. The test can be performed in approximately 30 minutes using a fully automated system, with sensitivity and specificity of 92.5% and 100%, respectively, compared to reverse transcription-quantitative polymerase chain reaction (RT-qPCR) using nasopharyngeal swabs as samples [8].

During winter 2020, Tokyo declared a state of emergency owing to the COVID-19 pandemic. At this time, we uniformly interviewed all newly admitted patients in our hospital for symptoms and history of contact with COVID-19 affected persons, and conducted quantitative SARS-CoV-2 antigen tests.

This study aimed to determine the following: the diagnostic accuracy of the SARS-CoV-2 antigen test; whether universal screening upon admission by a quantitative antigen test and a questionnaire for symptoms and exposure were effective to detect COVID-19 patients during the pandemic; and whether the antigen test was helpful in preventing a nosocomial outbreak in an acute-care hospital.

## Patients and methods

### Patients

This retrospective cohort study included patients aged 20 years or older admitted to Toranomon Hospital (819 beds, Tokyo, Japan) between December 3, 2020 and March 20, 2021. All patients underwent quantitative antigen testing using nasopharyngeal swabs within 24 hours of admission. Patients diagnosed with COVID-19 within the past 3 months, those who could not give their informed consent for the test, or those with difficulty obtaining appropriate specimens due to nasopharyngeal trauma, surgery, or epistaxis were excluded. We tested these patients using other methods, and they were not included in the study.

Patients with COVID-19 were defined as follows: 1) those with a positive SARS-CoV-2 NAAT result (RT-PCR or reverse transcription loop-mediated isothermal amplification; RT-LAMP) using nasopharyngeal swabs or sputum within 14 days of admission; or 2) those with a positive quantitative SARS-CoV-2 antigen test result on admission, symptoms or chest computed tomography (CT) imaging findings typical of COVID-19, and a COVID-19 diagnosis by consensus of at least two physicians, including an infectious disease or respiratory specialist.

### Admission screening and risk assessment

**Fig 1:**
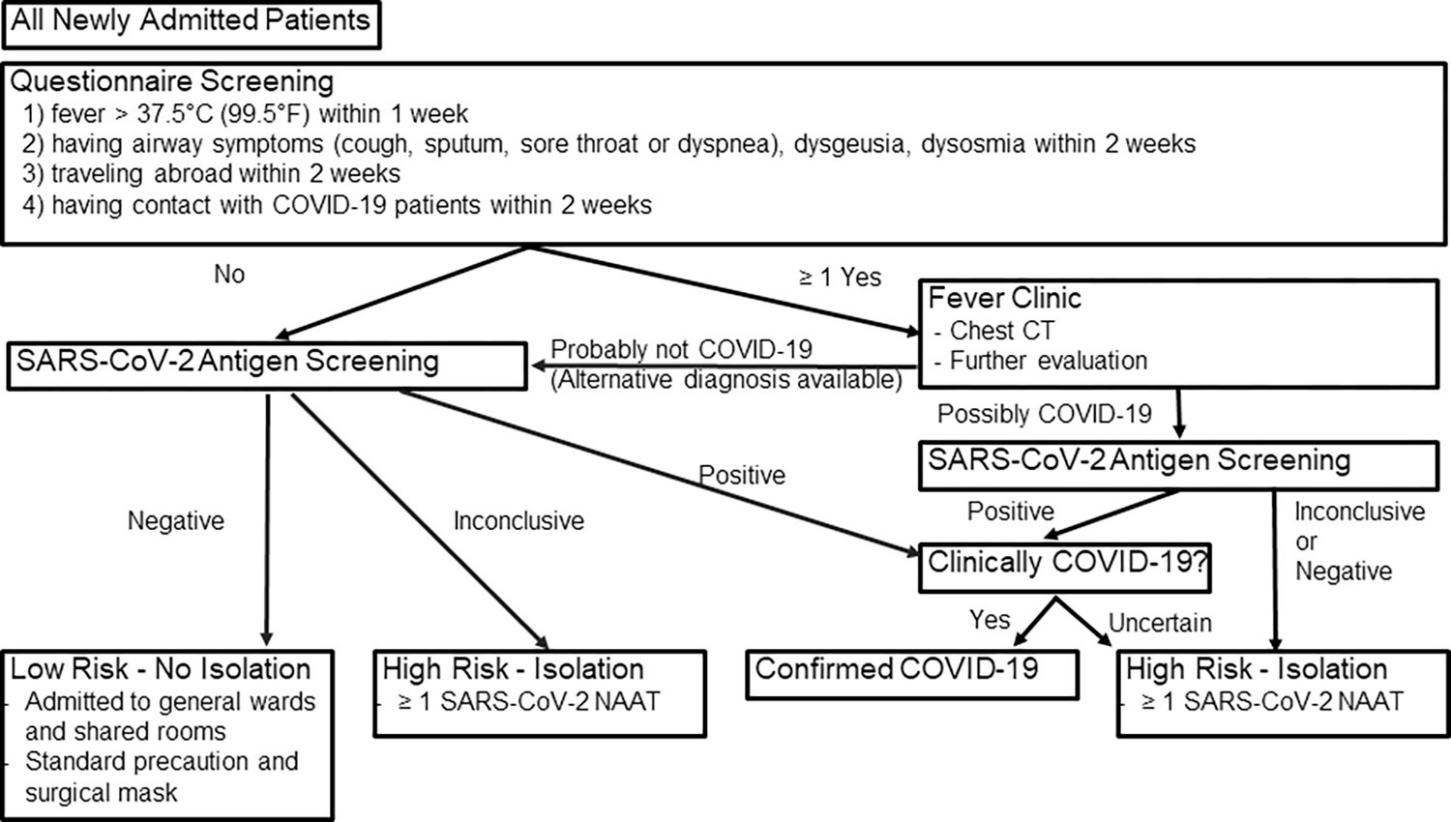
Admission screening and risk assessment strategy. All patients referred to the fever clinic underwent CT. All CT images were evaluated by radiologists. NAAT, Nucleic Acid Amplification Test; CT, computed tomography.

All admitted patients underwent SARS-CoV-2 antigen testing and questionnaire screening on admission (Fig 1).

We referred patients to the fever clinic if they answered “yes” to one or more questions. In addition to the routine SARS-CoV-2 antigen test on admission, we requested a chest CT scan and performed a NAAT if necessary. Then, a physician examined them to assess their risk for COVID-19. Patients with any of the following were classified as having “high risk” for COVID-19 and isolated in private rooms: 1) having contact with COVID-19 patients within 2 weeks, 2) fever clinic physician was unable to rule out COVID-19, and 3) positive or inconclusive admission antigen test. If none of the items on the questionnaire applied, or if the fever clinic physician considered the likelihood of COVID-19 to be low, the patient was classified to be at “low risk” for COVID-19. These patients underwent routine SARS-CoV-2 antigen testing on admission and were admitted to general wards and shared rooms.

### Isolation

On admission, at least two physicians, including an infectious disease or respiratory specialist, reviewed antigen-positive patients to confirm a COVID-19 diagnosis based on typical symptoms, CT images, and history of close contact. We admitted patients with confirmed COVID-19 to isolation wards.

Patients who were not diagnosed with COVID-19 at admission, but were classified as “high-risk” for COVID-19 by the screening (Fig 1) were isolated in private rooms according to the CDC’s provisional recommendations for infection control [9]. We continued isolation until we performed at least one NAAT after admission, and at least two physicians and an infectious disease specialist considered the possibility of COVID-19 sufficiently low. If we discharged the patient on the day of admission, a physician called or examined the patient 2 weeks later to confirm the absence of symptoms. The local health department also completed a follow-up.

Medical and microbiological records were retrospectively reviewed to confirm the results of the patient’s admission questionnaire, the SARS-CoV-2 antigen test, and NAAT.

The Research Ethics Review Committee of Toranomon Hospital approved this study (Certification No. 2192), and consent was substituted by presenting an opt-out document.

### Contacts

Contact, according to the Japanese National Institute of Infectious Diseases, is defined as follows [10]: contact between 2 days before the patient presents symptoms and the start of the quarantine; those who live together or spend long periods in contact with the patient; those who have examined, given care to, or nursed a patient, without taking adequate disease prevention measures; those who are likely to have touched something contaminated by viral particles present in coughs, sneezes, or body fluids of the patient; and those who have been in contact with a patient for more than 15 minutes within a distance of 1 meter without necessary preventive measures.

### Quantitative SARS-CoV-2 antigen test

All specimens were collected from the nasopharynx using cotton swabs, and immediately transported to the hospital laboratory in sterile containers for immediate testing. The antigen concentration was measured using LUMIPULSE SARS-CoV-2 Ag (Fujirebio, Inc., Tokyo, Japan), and the testing equipment was a LUMIPULSE G 1200 (Fujirebio, Inc., Tokyo, Japan). The test was performed according to the manufacturer’s guideline. Antigen concentration >10.00 pg/mL was considered positive for antigen quantification tests and a concentration between 1.00 pg/mL and 10.00 pg/mL was considered inconclusive. Although dilution retesting is recommended for antigen concentrations >5000 pg/mL, we considered the result as 5000 pg/mL because of the shortage of personal protective equipment, swabs and reagents, and the increased risk of infection to the laboratory technician.

### SARS-CoV-2 NAAT

Due to supply problems of reagents, nasopharyngeal swabs collected between December 3, 2020 and February 16, 2021 were tested in the hospital using RT-LAMP according to previous studies [11, 12], and nasopharyngeal swabs collected between February 16, 2021 and March 20, 2021 were tested using RT-qPCR. In all periods, RT-qPCR was used to test sputum in an external laboratory.

Nasopharyngeal swabs were soaked in 1–3 mL of saline solution immediately after collection, placed in sterile containers, and transported to the in-hospital laboratory for RT-qPCR testing within 2 hours of collection using the 2019 Novel Coronavirus COVID-19 Detection Kit (Shimadzu Corporation, Kyoto, Japan) according to the instructions provided [13]. Sputum was sent to BML, Inc. (Tokyo, Japan) for RT-qPCR testing [14].

### Outcomes

The primary outcome was the diagnostic performance (sensitivity and specificity) of the quantitative antigen test. The secondary outcome was the absence of a nosocomial outbreak of COVID-19 during the study period and subsequent 14-day observation period. In addition, to investigate whether there were patients with negative quantitative antigen tests at admission who developed COVID-19 during hospitalization, we analyzed cases in which the SARS-CoV-2 antigen test results at admission did not match the NAAT results after admission in the database. Moreover, among the cases that were antigen-positive or inconclusive, we compared the antigen concentrations between cases diagnosed with COVID-19 and patients without COVID-19.

### Statistical analysis

Statistical analyses were performed with R (The R Foundation for Statistical Computing) and EZR (Saitama Medical Center, Jichi Medical University, Saitama, Japan), which is a graphical user interface for R [15]. Categorical variables were compared using Fisher’s exact test. The Mann–Whitney U test or Welch’s t-test was used to analyze continuous variables. In all analyses, the level of significance was set at p = 0.05.

## Results

### Screening by antigen test

**Fig 2:**
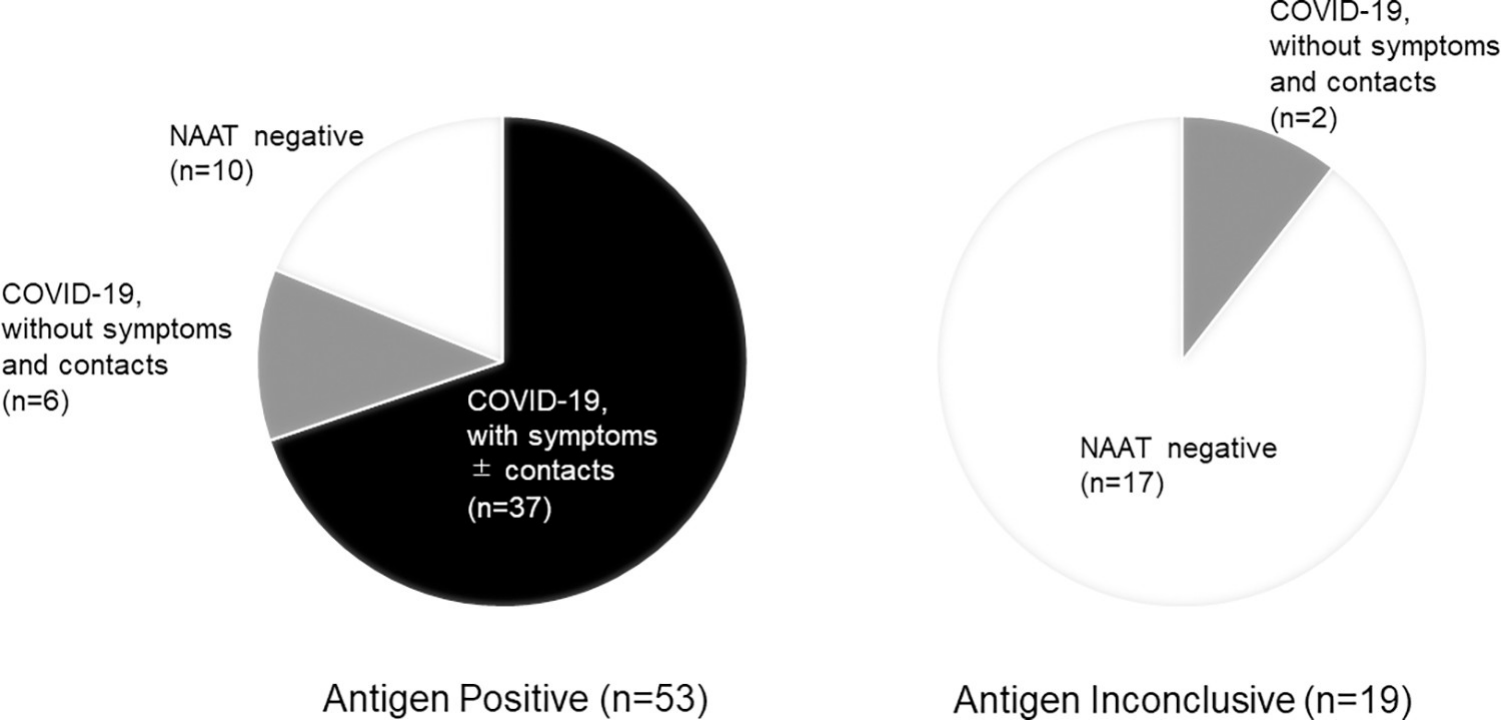
Screening by quantitative SARS-CoV-2 antigen test on admission. 43 patients with positive, two with inconclusive and two with negative (not shown in this figure) antigen test results on admission were finally diagnosed with COVID-19. Six patients with COVID-19 had neither symptoms nor contacts. COVID-19, coronavirus disease 2019; NAAT, Nucleic Acid Amplification Test.

A total of 5191 patients were included in the study, of which 53 were positive for quantitative antigen testing on admission, 19 were inconclusive, and 5119 were negative. Finally, 47 patients were diagnosed with COVID-19 based on either a positive NAAT result alone or a positive antigen-test result with a consistent clinical picture. Fig 2 shows the result of the antigen test and the final diagnosis in each group.

Of the 53 patients positive for quantitative antigen on screening, 37 were confirmed to have COVID-19 at the time of admission by typical symptoms and CT findings. The 16 patients who were antigen positive but not diagnosed with COVID-19 at the time of admission and all 19 patients with inconclusive results were isolated and underwent NAAT within 3 days. Among these, NAAT was positive in eight patients, and they were diagnosed with COVID-19; six patients with COVID-19 had neither symptoms nor contacts. The remaining 27 patients were NAAT-negative and not diagnosed with COVID-19. Of the 5119 patients with negative antigen test results on admission, two were confirmed to have COVID-19 (both NAAT positive), and 5117 patients were not diagnosed with COVID-19. The two who were later diagnosed with COVID-19 were isolated on admission based on the responses to the questionnaire.

Patient characteristics are shown in Table 1. Patients who tested positive for antigen were more likely to have a history of contact with patients with COVID-19, fever, pneumonia, and older age than those who had indeterminate results. The median isolation period was 8 days (range 3–20 days).

**Table 1:** Characteristics of patients with positive and inconclusive antigen test results.

	Total (N = 72)			COVID-19 Cases (N = 45)		
Result of Lumipulse®	Positive	Inconclusive	P value	Positive	Inconclusive	P value
**Total**	53/72 (73.6%)	19/72 (26.4%)		43/45 (95.6%)	2/45 (4.4%)	
**Median Age (range)**	67 (27–96)	74 (32–91)	0.10	63 (27–96)	66 (48–83)	0.92
**Sex, Male**	34/53 (64.2%)	11/19 (57.9%)	0.78	30/43 (69.8%)	2/2 (100%)	1.00
**Contact with patients with COVID-19**	13/53 (24.5%)	0/19 (0%)	0.02	12/43 (27.9%)	0/2(0%)	1.00
**Symptoms**						
Symptomatic	45/53 (84.9%)	11/19 (57.9%)	0.02	41/43 (95.3%)	2/2 (100%)	1.00
Fever	38/53 (71.7%)	6/19 (31.6%)	0.01	34/43 (79.1%)	1/2 (50%)	0.40
Airway Symptoms	30/53 (56.6%)	7/19 (36.8%)	0.18	28/43 (65.1%)	1/2 (50%)	1.00
Anosmia/Ageusia	0/53 (0%)	1/19 (5.3%)	0.26	0/43 (0%)	1/2 (50%)	0.04
**Median Days from Symptom Onset to Antigen Testing (range)**	3 (1–15)	3 (1–14)	0.84	4 (1–15)	9 (4–14)	0.55
**Abnormal CT findings suggestive of Pneumonia** ^**a**^	32/53 (60.4%)	5/19 (26.3%)	0.02	30/43 (69.8%)	1/2 (50%)	0.53
**Comorbidities**						
Diabetes Mellitus	12/53 (22.6%)	5/19 (26.3%)	0.76	11/43 (25.6%)	0/2 (0%)	1.00
Hypertension	17/53 (32.1%)	5/19 (26.3%)	0.78	14/43 (32.6%)	0/2 (0%)	1.00
Heart Disease	10/53 (18.9%)	5/19 (26.3%)	0.52	8/43 (18.6%)	0/2 (0%)	1.00
Chronic Kidney Disease	9/53 (17.0%)	1/19 (5.3%)	0.27	7/43 (16.3%)	0/2 (0%)	1.00
Liver Disease	4/53 (7.5%)	3/19 (15.8%)	0.37	4/43 (9.3%)	0/2 (0%)	1.00
Hematological Malignancy	2/53 (3.8%)	2/19 (10.5%)	0.28	1/43 (2.3%)	0/2 (0%)	1.00
Solid Tumor	12/53 (22.6%)	8/19 (42.1%)	0.14	9/43 (20.9%)	0/2 (0%)	1.00
Asthma	7/53 (13.2%)	1/19 (5.3%)	0.67	6/43 (14.0%)	0/2 (0%)	1.00
COPD/ILD	3/53 (5.7%)	3/19 (15.8%)	0.18	2/43 (4.7%)	0/2 (0%)	1.00
Transplant	4/53 (7.5%)	1/19 (5.3%)	1.00	3/43 (7.0%)	0/2 (0%)	1.00
Neurological Disease/Dementia	7/53 (13.2%)	4/19 (21.1%)	0.47	6/43 (14.0%)	0/2 (0%)	1.00
Cerebrovascular Disease	5/53 (9.4%)	3/19 (15.8%)	0.43	4/43 (9.3%)	0/2 (0%)	1.00
Corticosteroid Use	9/53 (17.0%)	2/19 (10.5%)	0.72	5/43 (11.6%)	0/2 (0%)	1.00
History of Smoking	16/53 (30.2%)	7/19 (36.8%)	0.58	14/43 (32.6%)	0/2 (0%)	1.00
Immunocompromised Status	2/53 (3.8%)	1/19 (5.3%)	1.00	2/43 (4.7%)	0/2 (0%)	1.00
Overweight (BMI >25)	13/53 (24.5%)	1/19 (5.3%)	0.09	13/43 (30.2%)	0/2 (0%)	1.00
Elderly (Age ≥65)	26/53 (49.1%)	15/19 (78.9%)	0.03	21/43 (48.8%)	1/2 (50%)	1.00

^a^ All images were evaluated by diagnostic radiology specialists.

COPD, Chronic Obstructive Pulmonary Disease; ILD, Interstitial Lung Disease; BMI, Body Mass Index.

The highest 7-day moving average of infected persons in Tokyo during the period was 1861.1/14.03 million, and the SARS-CoV-2 test positivity rate was 14.5% [16].

Table 2 shows the results of the antigen test on admission and diagnosis of COVID-19 within 14 days after hospitalization. The sensitivity and specificity of the antigen-quantification test (positive or inconclusive) for diagnosing COVID-19 at admission was 0.957 (95% confidence interval [CI], 0.855–0.995) and 0.995 (95% CI, 0.992–0.997), respectively. In addition, the sensitivity and specificity of the antigen-quantification test for diagnosing COVID-19 at admission was 0.956 (95% CI, 0.849–0.995) and 0.994 (95% CI, 0.992–0.996), respectively, when limiting the definition of patients with COVID-19 as 1) those with a positive SARS-CoV-2 RT-PCR result (those with a positive RT-LAMP result were excluded) within 14 days of admission; or 2) those with a positive quantitative SARS-CoV-2 antigen test result on admission, symptoms or CT imaging findings typical of COVID-19, and a COVID-19 diagnosis by consensus of at least two physicians (S1 Table). Sensitivity analysis of patients who underwent NAAT is shown in S2 Table.

**Table 2:** Results of SARS-CoV-2 antigen test on admission and diagnosis of COVID-19 within 14 days after hospitalization.

		Diagnosis	
		COVID-19	Non-COVID
Antigen	+ / ±	45	27
	**−**	2	5117

+, Positive. ±, Inconclusive. −, Negative.

### Screening using a combination of the quantitative antigen test and a questionnaire

Of the 5191 patients included in the study, 619 were classified as “high-risk” based on the results of quantitative antigen test or questionnaire screening on admission. Of these, 47 had a confirmed diagnosis of COVID-19, and 572 did not. There were 4572 patients with negative admission quantitative antigen test results, who were categorized as “low-risk” after screening questionnaires and were not isolated at the time of admission. None of them were diagnosed with COVID-19 in the subsequent 14 days of admission.

### Discrepancy between admission screening antigen test and NAAT results after hospitalization

Among patients finally diagnosed with COVID-19, the results of antigen test on admission and NAAT after hospitalization were inconsistent in five patients. Of these, two had negative antigen tests on admission, and both were close contacts of patients with COVID-19; two patients had inconclusive results; and one patient had a positive antigen test on admission, and two consequent false-negative NAAT results, followed by a positive NAAT result.

### Occurrence of nosocomial outbreaks of COVID-19

No nosocomial outbreaks occurred during the study period and the subsequent observation period.

### Comparison of antigen concentrations between patients with and without COVID-19

**Fig 3:**
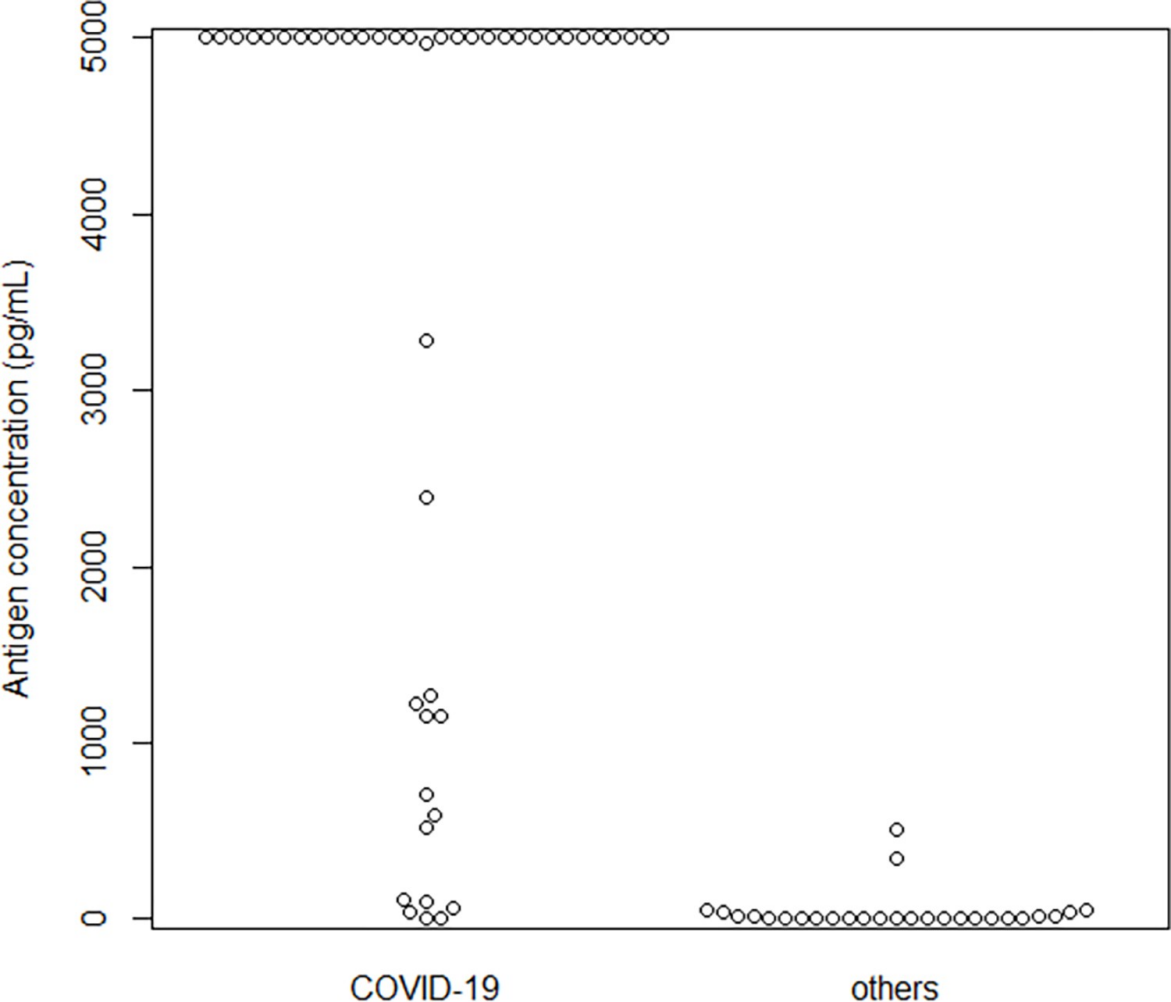
Comparison of antigen concentrations between patients with and without COVID-19.

Among the antigen-positive and inconclusive cases, there was a significant difference in the antigen concentrations between those with and without confirmed COVID-19 (p < 0.001) (Fig 3).

## Discussion

To the best of our knowledge, this is the first clinical study to use the LUMIPULSE SARS-CoV-2 antigen test for universal screening of all hospitalized patients. There were three new findings in this study. First, LUMIPULSE SARS-CoV-2 Ag is a highly accurate diagnostic test for COVID-19, even as a universal screening test on admission. Second, the combination of quantitative antigen screening and simple questionnaire screening on admission did not miss capturing several COVID-19 patients with false-negative antigen test results. Third, no nosocomial SARS-CoV-2 infections occurred during the study period after implementation of this admission screening strategy.

In this study, the sensitivity of the quantitative antigen test alone at the time of admission was high enough to be reliable. This result was similar to the 92.5% sensitivity and 100% specificity reported in a previous study in which symptomatic patients, asymptomatic contacts of patients with COVID-19, and those who had traveled abroad were tested with LUMIPULSE SARS-CoV-2 Ag [8].

The minimum antigen concentration in patients with confirmed COVID-19 was 3.55 pg/mL, which was higher than the previously reported cutoff value of 1.34 pg/mL [17]. On the other hand, there were 10 patients with antigen-positive and negative NAAT results were not diagnosed with COVID-19 (18.8% of all antigen-positive cases on admission), and the maximum antigen concentration was 508.05 pg/mL. Probable causes were contamination with blood components or highly viscous impurities, and specimens evaluated immediately after a strongly positive specimen, which affected the measured values. By addressing these problems in specimen processing, the number of false positives decreased. However, false positives may still occur, which highlights the importance of clinical evaluation.

Furthermore, among the 5119 antigen-negative patients, NAAT was positive in two patients who were later diagnosed with COVID-19, suggesting that the utility of antigen screening alone may be limited. In addition, with questionnaire screening alone, patients who do not report minor symptoms or contacts or those prone to symptoms due to an underlying disease, may be overlooked and not isolated. In our study, in some patients with COVID-19 who were asymptomatic on admission, imaging after positive antigen-test result revealed silent pneumonia. Universal antigen testing may have helped prevent nosocomial infection because patients who would not have been identified based on symptom screening were quickly identified and isolated. Therefore, antigen testing combined with a questionnaire may reduce the possibility of missing false-negatives and prevent the spread of infection in the hospital. In fact, at our institution, we prevented spread because we could find contacts of patients with COVID-19 through the screening questionnaire and isolate all patients with false-negative antigen-test results. Consequently, antigen screening alone might not be enough, and it is crucial to conduct both antigen and questionnaire screening.

This study had some limitations. First, it was a retrospective, single-center study. Second, some cases of COVID-19 were diagnosed clinically based on positive antigen-test results and clinical findings consistent with COVID-19. Moreover, due to the limited supply of reagents, NAAT was not performed in all cases and methods of NAAT were not standardized. The sensitivity of antigen test was lower in the cohort diagnosed based on NAAT than in the cohort diagnosed based on the combination strategy; the 95% confidence interval was wide due to the small number of cases diagnosed with COVID-19 based on NAAT. We need to continue the study with more cases with positive NAAT results. However, most patients who did not undergo NAAT because they were antigen-positive and had a consistent clinical picture had antigen concentrations of 5000 pg/mL or higher, with a minimum value of 66.56 pg/mL. This value is much higher than the minimum antigen concentration in PCR-positive samples reported in previous studies [8, 18]. In addition, since there could be false-negative NAAT results, it would be practical to diagnose patients with COVID-19 based on positive antigen-test results and a consistent clinical picture. And also, the sensitivity and specificity of the antigen test were still high even if patients diagnosed with positive RT-LAMP results were excluded from the diagnosis of COVID-19 in this population. Third, in areas with a high prevalence of COVID-19, this screening may not be clinically useful. Although, during the subsequent epidemic period, the SARS-CoV-2 test positivity rate in Tokyo was as high as 21.3%, and the 7-day moving average of infected persons reached 4923.4/14.03 million [16], no nosocomial infections have occurred in our hospital using this strategy to date. This experience suggests the strategy may be useful in settings with high disease prevalence.

Lastly, since the study period was during the COVID-19 wave caused by the conventional and alpha strains of SARS-CoV-2, it is unclear whether these results will be applicable for other mutant strains, and further studies are needed.

## Conclusion

The LUMIPULSE SARS-CoV-2 Ag is a highly diagnostic test, and when used in conjunction with a simple questionnaire to assess symptoms and contacts, it is expected to identify antigen-negative patients with COVID-19 on admission. Consequently, this screening strategy prevents the nosocomial spread of COVID-19 and protects hospitalized patients who are prone to severe illness due to underlying medical conditions.

## Supporting information

S1 Table Results of SARS-CoV-2 antigen test on admission and diagnosis of COVID-19 within 14 days after hospitalization, excluding patients diagnosed based on RT-LAMP. S1 Table shows the results when we limited the definition of patients with COVID-19 as 1) those with a positive SARS-CoV-2 RT-PCR result within 14 days of admission; or 2) those with a positive quantitative SARS-CoV-2 antigen test result on admission, symptoms or CT imaging findings typical of COVID-19, and a COVID-19 diagnosis by consensus of at least two physicians. Patients diagnosed based on positive RT-LAMP results were excluded. The sensitivity and specificity of the antigen-quantification test (positive or inconclusive) for diagnosing COVID-19 at admission was 0.956 (95% CI, 0.849–0.995) and 0.994 (95% CI, 0.992–0.996), respectively. RT-PCR, reverse transcription polymerase chain reaction; RT-LAMP, reverse transcription loop-mediated isothermal amplification; CI, confidence interval, +, Positive; ±, Inconclusive; −, Negative.
(DOCX)

S2 Table Comparison of the antigen test and NAAT results. **A. Any NAAT performed.** NAAT includes RT-PCR and RT-LAMP. The sensitivity of a positive or inconclusive antigen test on admission to a positive NAAT test is 0.818 (95% CI, 0.482–0.977) and specificity was 0.946 (95% CI, 0.925–0.962). **B. RT-PCR.** The sensitivity of a positive or inconclusive antigen test on admission to a positive RT-PCR was 0.714 (95% CI, 0.290–0.963) and specificity was 0.868 (95% CI, 0.813–0.912). **C. RT-LAMP.** The sensitivity of a positive or inconclusive antigen test on admission to a positive RT-LAMP was 1.000 (95% CI, 0.194–1.000) and specificity was 0.950 (95% CI, 0.926–0.968). **D. Not tested by NAAT.** Clinical diagnosis was made based on positive antigen test results (not including inconclusive) with symptoms and CT imaging findings typical of COVID-19 by a consensus of two or more experts. NAAT was performed if the diagnosis could not be clinically confirmed (thus, such patients are not included in this cohort). The sensitivity of a positive antigen test on admission to a clinical diagnosis of COVID-19 was 1.000 (95% CI, 0.865–1.000) and specificity was 1.000 (95% CI, 0.999–1.000). NAAT, Nuclear Acid Amplification Test; RT-qPCR, reverse transcription-quantitative polymerase chain reaction; RT-LAMP, reverse transcription loop-mediated isothermal amplification; CI, confidence interval, +, Positive; ±, Inconclusive; −, Negative.
(DOCX)
