# Supplementary material for: Universal admission screening for COVID-19 using quantitative antigen testing and questionnaire screening to prevent nosocomial spread
Source: PLoS One. 2022 Nov 10;17(11):e0277426. doi: 10.1371/journal.pone.0277426 (PMC9648767; doi:10.1371/journal.pone.0277426)
Supplement: S1 Table — shows the results when we limited the definition of patients with COVID-19 as 1) those with a positive SARS-CoV-2 RT-PCR result within 14 days of admission; or 2) those with a positive quantitative SARS-CoV-2 antigen test result on admission, symptoms or CT imaging findings typical of COVID-19, and a COVID-19 diagnosis by consensus of at least two physicians. Patients diagnosed based on positive RT-LAMP results were excluded. The sensitivity and specificity of the antigen-quantification test (positive or inconclusive) for diagnosing COVID-19 at admission was 0.956 (95% CI, 0.849–0.995) and 0.994 (95% CI, 0.992–0.996), respectively. RT-PCR, reverse transcription polymerase chain reaction; RT-LAMP, reverse transcription loop-mediated isothermal amplification; CI, confidence interval, +, Positive; ±, Inconclusive; −, Negative. (DOCX) [file pone.0277426.s001.docx]

**S1 Table. Results of SARS-CoV-2 antigen test on admission and diagnosis of COVID-19 within 14 days after hospitalization, excluding patients diagnosed based on RT-LAMP.**

|  |  | Diagnosis | |
| --- | --- | --- | --- |
|  |  | COVID-19 | Non-COVID-19 |
| Antigen | + / ± | 43 | 26 |
|  | **−** | 2 | 4663 |

S1 Table shows the results when we limited the definition of patients with COVID-19 as 1) those with a positive SARS-CoV-2 RT-PCR result within 14 days of admission; or 2) those with a positive quantitative SARS-CoV-2 antigen test result on admission, symptoms or CT imaging findings typical of COVID-19, and a COVID-19 diagnosis by consensus of at least two physicians. Patients diagnosed based on positive RT-LAMP results were excluded. The sensitivity and specificity of the antigen-quantification test (positive or inconclusive) for diagnosing COVID-19 at admission was 0.956 (95% CI, 0.849–0.995) and 0.994 (95% CI, 0.992–0.996), respectively.

RT-PCR, reverse transcription polymerase chain reaction; RT-LAMP, reverse transcription loop-mediated isothermal amplification; CI, confidence interval, +, Positive; ±, Inconclusive; −, Negative.
