# Supplementary material for: Universal admission screening for COVID-19 using quantitative antigen testing and questionnaire screening to prevent nosocomial spread
Source: PLoS One. 2022 Nov 10;17(11):e0277426. doi: 10.1371/journal.pone.0277426 (PMC9648767; doi:10.1371/journal.pone.0277426)
Supplement: S2 Table — A. Any NAAT performed. NAAT includes RT-PCR and RT-LAMP. The sensitivity of a positive or inconclusive antigen test on admission to a positive NAAT test is 0.818 (95% CI, 0.482–0.977) and specificity was 0.946 (95% CI, 0.925–0.962). B. RT-PCR. The sensitivity of a positive or inconclusive antigen test on admission to a positive RT-PCR was 0.714 (95% CI, 0.290–0.963) and specificity was 0.868 (95% CI, 0.813–0.912). C. RT-LAMP. The sensitivity of a positive or inconclusive antigen test on admission to a positive RT-LAMP was 1.000 (95% CI, 0.194–1.000) and specificity was 0.950 (95% CI, 0.926–0.968). D. Not tested by NAAT. Clinical diagnosis was made based on positive antigen test results (not including inconclusive) with symptoms and CT imaging findings typical of COVID-19 by a consensus of two or more experts. NAAT was performed if the diagnosis could not be clinically confirmed (thus, such patients are not included in this cohort). The sensitivity of a positive antigen test on admission to a clinical diagnosis of COVID-19 was 1.000 (95% CI, 0.865–1.000) and specificity was 1.000 (95% CI, 0.999–1.000). NAAT, Nuclear Acid Amplification Test; RT-qPCR, reverse transcription-quantitative polymerase chain reaction; RT-LAMP, reverse transcription loop-mediated isothermal amplification; CI, confidence interval, +, Positive; ±, Inconclusive; −, Negative. (DOCX) [file pone.0277426.s002.docx]

**S2 Table.** **Comparison of the antigen test and NAAT results**

1. **Any NAAT performed**

|  |  | NAAT | |
| --- | --- | --- | --- |
|  |  | + | − |
| Antigen | + / ± | 9 | 36 |
|  | **−** | 2 | 625 |

NAAT includes RT-PCR and RT-LAMP. The sensitivity of a positive or inconclusive antigen test on admission to a positive NAAT test is 0.818 (95% CI, 0.482–0.977) and specificity was 0.946 (95% CI, 0.925–0.962).

1. **RT-PCR**

|  |  | RT-PCR | |
| --- | --- | --- | --- |
|  |  | + | − |
| Antigen | + / ± | 5 | 26 |
|  | **−** | 2 | 171 |

The sensitivity of a positive or inconclusive antigen test on admission to a positive RT-PCR was 0.714 (95% CI, 0.290–0.963) and specificity was 0.868 (95% CI, 0.813–0.912).

1. **RT-LAMP**

|  |  | RT-LAMP | |
| --- | --- | --- | --- |
|  |  | + | − |
| Antigen | + / ± | 3 | 24 |
|  | **−** | 0 | 454 |

The sensitivity of a positive or inconclusive antigen test on admission to a positive RT-LAMP was 1.000 (95% CI, 0.194–1.000) and specificity was 0.950 (95% CI, 0.926–0.968).

1. **Not tested by NAAT**

|  |  | COVID-19 Clinical Diagnosis | |
| --- | --- | --- | --- |
|  |  | COVID-19 | Non-COVID |
| Antigen | + | 38 | 0 |
|  | **−** | 0 | 4492 |

Clinical diagnosis was made based on positive antigen test results (not including inconclusive) with symptoms and CT imaging findings typical of COVID-19 by a consensus of two or more experts. NAAT was performed if the diagnosis could not be clinically confirmed (thus, such patients are not included in this cohort). The sensitivity of a positive antigen test on admission to a clinical diagnosis of COVID-19 was 1.000 (95% CI, 0.865–1.000) and specificity was 1.000 (95% CI, 0.999–1.000).

NAAT, Nuclear Acid Amplification Test; RT-qPCR, reverse transcription-quantitative polymerase chain reaction; RT-LAMP, reverse transcription loop-mediated isothermal amplification; CI, confidence interval, +, Positive; ±, Inconclusive; −, Negative.
